# Structural and Functional Characterization of Human Peripheral Nervous System Myelin Protein P2

**DOI:** 10.1371/journal.pone.0010300

**Published:** 2010-04-22

**Authors:** Viivi Majava, Eugenia Polverini, Alberto Mazzini, Rahul Nanekar, Wiebke Knoll, Judith Peters, Francesca Natali, Peter Baumgärtel, Inari Kursula, Petri Kursula

**Affiliations:** 1 Department of Biochemistry, University of Oulu, Oulu, Finland; 2 Department of Physics, University of Parma, Parma, Italy; 3 Institut Laue-Langevin, Grenoble, France; 4 University Joseph Fourier, Grenoble, France; 5 Institut de Biologie Structurale, Grenoble, France; 6 Consiglio Nazionale delle Richerche – Operative Group in Grenoble, Grenoble, France; 7 BESSY II, Helmholtz-Zentrum Berlin, Berlin, Germany; 8 Centre for Structural Systems Biology, Helmholtz Centre for Infection Research, Braunschweig, Germany; 9 German Electron Synchrotron, University of Hamburg, Hamburg, Germany; University of Queensland, Australia

## Abstract

The myelin sheath is a tightly packed multilayered membrane structure insulating selected axons in the central and the peripheral nervous systems. Myelin is a biochemically unique membrane, containing a specific set of proteins. In this study, we expressed and purified recombinant human myelin P2 protein and determined its crystal structure to a resolution of 1.85 Å. A fatty acid molecule, modeled as palmitate based on the electron density, was bound inside the barrel-shaped protein. Solution studies using synchrotron radiation indicate that the crystal structure is similar to the structure of the protein in solution. Docking experiments using the high-resolution crystal structure identified cholesterol, one of the most abundant lipids in myelin, as a possible ligand for P2, a hypothesis that was proven by fluorescence spectroscopy. In addition, electrostatic potential surface calculations supported a structural role for P2 inside the myelin membrane. The potential membrane-binding properties of P2 and a peptide derived from its N terminus were studied. Our results provide an enhanced view into the structure and function of the P2 protein from human myelin, which is able to bind both monomeric lipids inside its cavity and membrane surfaces.

## Introduction

The myelin sheath is a multilayered membrane surrounding selected axons in the vertebrate nervous system, formed by the highly specialized plasma membrane of a myelinating glial cell. Biochemically, myelin is formed of roughly 70–85% lipid, with a high content of cholesterol [1], and only 15–30% of protein, especially myelin-specific proteins. Peripheral myelin protein 2 (P2) constitutes up to 15% of total protein of peripheral nervous system (PNS) myelin [2]. P2 is also present in small amounts in central nervous system (CNS) myelin, being abundant in spinal cord and brain stem myelin [3]–[5]. The amount of P2, however, varies between different species, different regions of the nervous system and from fiber to fiber [6]. P2 is localized at the major dense line of myelin sheaths, and therefore, it is likely to play a similar structural role in peripheral myelin as the myelin basic protein (MBP) in both the CNS and PNS; the two proteins are, however, genetically and structurally unrelated. As a structural protein, P2 is thought to stabilize the myelin membranes. Our recent neutron scattering experiments, indeed, have shown that upon binding to lipid bilayers, P2 decreases the lipid dynamics [7].

By X-ray diffraction, it has been determined that myelin in some species has wider major dense lines when there are large amounts of P2 [8], [9]. Human P2 is a basic cytosolic 131-amino-acid 14.5-kDa protein, with a +10 net basic charge. Bovine peripheral myelin P2 crystal structure was the first to be solved for a full-length myelin protein [10], [11]. Also the equine P2 crystal structure was solved more recently [12]. The proteins were purified from spinal cord in these studies.

Structurally, P2 belongs to the family of cytoplasmic fatty acid binding proteins (FABPs) that has evolved by successive gene duplications; the members of this family can solubilize and transport fatty acids and retinoids in the cytoplasm [13]–[15]. Hence, P2 might have functions in myelin assembly and turnover. However, the transport role of the FABPs has tissue dependent differences in ligand specificity and binding affinity, and consequently, in fatty acid metabolism [16].

P2 fragments are known to cause infammatory demyelination in experimental allergic neuritis (EAN), an animal model of Guillain-Barrè syndrome (GBS) [17]–[19]. Also antibodies to P2 protein and peptides have been found in GBS patients [20], [21]. As P2 is not known to be exposed at the cell surface, antibodies against it are unlikely to initiate disease, and antibodies directed against myelin proteins are likely to be directly involved in demyelination in only a small proportion of GBS cases [22]. Chronic infammatory demyelinating polyradiculoneuropathy CIDP, assumed to be the chronic counterpart of acute GBS [18], is an acquired immune-mediated infammatory disorder of the PNS, with a probable autoimmune pathogenesis. In CIDP, P2 was found to be the antigen most likely involved in the immune responses due to nerve damage [23].

Traditionally, myelin proteins have been purified directly from vertebrate nervous tissues. We use recombinant techniques to produce myelin proteins, which enables the controlled production of homogeneous samples with or without desired mutations. Here, we purified recombinant human P2 in large scale and solved its crystal structure. The structure was also characterized by small-angle X-ray scattering (SAXS) and synchrotron radiation circular dichroism spectroscopy (SRCD). Structure-based analyses of lipid ligand binding were also carried out by computational and fluorescence methods, and the membrane-binding properties were studied using membrane mimics.

## Materials and methods

### Preparation of the human P2 expression vector

The cDNA of human P2 was purchased from RZPD (German Resource Center for Genome Research). Primers for Gateway cloning (Invitrogen) were from Oligomer. PCR reactions were performed in two steps. The primers for the first PCR reaction were 5′AAAAAGCAGGCTCTGAGAATCTTTATTTTCAGGGCATGAGCAACAAATTCCG 3′ and 5′ AGAAAGCTGGGTTCAGACCTTCTCATAGATTCTG 3′. In the second PCR reaction, the forward primer 5′ GGGGACAAGTTTGTACAAAAAAGCAGGCT 3′ and the reverse primer 5′ GGGGACCACTTTGTACAAGAAAGCTGGGT 3′ were used to append sites for homologous recombination at the end of the PCR products. PCR was done using the Phusion High-Fidelity DNA Polymerase (Finnzymes), according to the manufacturer's instructions. PCR products were purified after both reactions using the polyethylene glycol (PEG) protocol (Invitrogen, Gateway manual) to remove small DNA fragments. The Gateway BP and LR Clonase II Enzyme Mix kits (Invitrogen) were used for cloning, according to the manufacturer's instructions. Briefly, the PCR product was recombined with the donor vector pDONR221 (Invitrogen) to create the entry clone. The products were transformed into *E. coli* DH5α cells, and plasmids were purified from 3-ml cultures, originating from single colonies, and sequenced. The pTH27 plasmid, coding for an N-terminal His_6_-tag [24], was used as the destination vector; recombination of the destination vector with the entry clone yielded the expression clone. After transformation into DH5α cells, colonies were picked, and plasmid isolation and sequencing carried out. All cloning steps were performed as described in the Gateway manual (Invitrogen), but in downscaled reaction volumes (a total volume of 5.0 µl, consisting of 3.0 µl of TE buffer pH 8.0, 0.5 µl of acceptor vector, and 0.5 µl donor DNA, and 1 µl of Gateway BP or LR Clonase II enzyme mix).

### Recombinant protein expression

The P2 expression vector was transformed into *E. coli* Rosetta(DE3) (Novagen). Overnight cultures were grown in 10 ml of LB with 100 µg/ml ampicillin and 34 g/ml chloramphenicol at +37°C. One liter of ZYM-5052 autoinduction medium [25] was then inoculated with the overnight culture, and two slightly different protocols for large-scale expression were used. For the protein eventually used for crystallization, the cells were grown at +20°C. After 16 h, 0.2 mM isopropyl-β-D-thiogalactoside and additional 100 µg/ml ampicillin and 17 µg/ml chloramphenicol were added. This addition was done to safeguard against loss of lactose and antibiotics during the prolonged culture. The cells were harvested after 36 h by centrifugation and stored at −70°C. For the other batches of P2, the cells were grown first for 4 h at +37°C and thereafter for 48 h at +18°C before harvesting.

### Protein purification

20 ml of lysis buffer (50 mM Na phosphate pH 8.0, 300 mM NaCl, 10 mM imidazole) and one Complete Mini EDTA-free protease inhibitor tablet (Roche) were added per g of cells. The suspension was mixed at +4°C and vortexed. Cells were disrupted by sonication, and cell debris was removed by centrifugation at 20000 g for 45 min at +4°C. The supernatant was applied onto Ni-NTA matrix (Qiagen) equilibrated with the lysis buffer. The matrix was washed with 50 mM Na-phosphate pH 8.0, 300 mM NaCl, 20 mM imidazole, and elution of His-P2 was done using 50 mM Na-Phosphate pH 8.0, 300 mM NaCl, 250 mM imidazole, following the absorbance at 280 nm.

The fractions were checked on SDS-PAGE gel and pooled. The concentration was measured at 280 nm, and recombinant TEV protease [26] was added at an approximate molar ratio of 1∶30 to His-P2. Cleavage was carried out for 24 h at +4°C, the sample was concentrated in an Amicon Ultra-15 3K centrifugal filter device (Millipore), and imidazole was removed using a PD-10 column (GE Healthcare); this allowed for the removal of uncleaved His-tagged protein by another passage through Ni-NTA in the case of incomplete cleavage. The completeness of the cleavage was analyzed on SDS-PAGE. The final purification step was done by gel filtration on a Superdex 75 16/60 (GE Healthcare) column in 20 mM HEPES pH 7.5, 50 mM NaCl, 10% glycerol.

For one batch, TEV cleavage was not carried out; rather, the protein was dialyzed to remove excess imidazole. The His-tagged protein was used only for three experiments: for a comparison of tagged and non-tagged protein by SRCD, and the fluorescence assays of DPC interaction (alongside with the non-tagged protein) and cholesterol binding.

### Synchrotron radiation circular dichroism spectroscopy

SRCD spectra for untagged P2 were collected on beamline CD1 of the ASTRID storage ring at the University of Århus, Denmark. P2 was dialyzed into 20 mM sodium phosphate (pH 7.4). All sample and baseline spectra were collected in quartz cells 3 times over the wavelengths 280 to 170 nm. The data were processed using CDtool [27] and analyzed for secondary structure using Dichroweb [28].

SRCD measurements were also performed at the BESSY beamline 3m-NIM-C in a similar manner, essentially as previously described [29]. In this case, the samples included His-tagged and untagged P2, as well as a peptide from the N terminus of human P2. The P2 samples were measured both in solution and as a dried film, and the peptide in the presence and absence of different membrane-mimicking compounds. The solution samples were measured at concentrations between 0.6–1 mg/ml, in a quartz cuvette of 100 µm pathlength. For measuring dry-phase SRCD data, the optimal condition was found to be a protein concentration between 0.5–1 mg/ml; one µl of this solution was pipetted into the centre of a calcium fluoride circular cuvette. The drop was allowed to evaporate standing still in the flow cabinet (a few minutes), after which the cuvette was further incubated under vacuum at +37°C for a few minutes. The cuvette was then inserted into the sample holder; the circular cuvette was always in the same orientation with respect to the incoming beam. For measuring the dry phase spectra, the sample chamber was extensively flushed with nitrogen gas, to remove traces of oxygen that otherwise caused high absorbance at 165 nm. Data processing was carried out using a local SRCD extension of IgorPro (Wavemetrics) available at BESSY.

In addition to SRCD measurements, a regular CD spectrometer was used to obtain a melting curve for P2. Briefly, the CD signal was followed at 218 nm as a function of temperature (+20 to +95°C, slope +1°C/min) in a Jasco J-715 Spectropolarimeter. The protein concentration was 0.12 mg/ml and a 1-mm quartz cuvette was used.

### Small-angle X-ray scattering

For SAXS, P2 was dialyzed into a buffer containing 50 mM HEPES (pH 7.5), 150 mM NaCl, and 10% glycerol. SAXS data were measured at concentrations up to 4 mg/ml on the X33 synchrotron radiation beamline at EMBL-Hamburg, DESY. Data processing and analysis were carried out using programs of the ATSAS package [30], essentially as previously described [29].

### Fluorescence titration of peptide-detergent interactions

A peptide from the N-terminus of P2 (residues 1–10, SNKFLGTWKL, purchased from SBS Genetech, China) was identified as a putative membrane binding site using Amphipaseek [31]. Since the peptide contains a Trp residue, intrinsic fluorescence spectroscopy was used to study its binding to dodecylphosphocholine (DPC) micelles. The assay was performed at +30°C using the Tecan Infinite M200 apparatus, in 96-well plates. The peptide (10 µg/ml) was incubated at different concentrations of DPC in 10 mM KPO_4_ buffer (pH 7), and excitation was done at 295 nm. Emission spectra were measured from 320 to 400 nm. Similarly, the recombinant human P2 protein, with and without His-tag, was analysed, at a concentration of 30 µg/ml.

### Crystallization, data collection, structure determination and refinement

The untagged protein was concentrated to 15 mg/ml and crystallized at +4°C using a mother liquor containing 280 mM Na phosphate, 100 mM Tris –HCl (pH 8.5), and 37% PEG4000. Prior to data collection, a crystal was flash-frozen in liquid nitrogen. Data were collected remotely using mxCUBE, at the ID14-1 beamline of ESRF Grenoble. The data were processed and scaled using XDS [32] and XDSi [33]. The data processing statistics are shown in Table 1. The structure was solved by molecular replacement using Phaser software [34] and the equine P2 structure (PDB entry 1YIV, [12]); the crystal form had one monomer per asymmetric unit. The structure was refined using Refmac5 [35] and phenix.refine [36] and built and analyzed with Coot [37]. The coordinates and structure factors were deposited at the Protein Data Bank with the accession code 2WUT. Images describing structural information were made with PyMol (www.pymol.org) and CCP4mg [38].

**Table 1:** Crystallographic data collection and structure refinement.

**Data collection**	
Space group	P4_1_2_1_2
Unit cell dimensions (Å)	a = 66.3, b = 66.3, c = 101.2
Resolution (Å)	20-1.85 (1.90-1.85)
R_merge_ (%)	6.0 (64.0)
<I/σI>	24.2 (2.3)
Completeness (%)	98.5 (87.8)
Redundancy	6.6 (4.7)
**Refinement**	
R_cryst_/R_free_ (%)	16.6/20.9
RMS Deviations from ideal values	
Bond lengths (Å)	0.007
Bond angles (°)	1.0
Average *B* factor/Wilson *B* (Å^2^)	28.9/23.8
Ramachandran plot (%)	
Preferred regions	99.1
Allowed regions	0.9
Outliers	0

The values in parentheses correspond to the high-resolution shell.

### Computational docking of lipids inside human P2

For docking simulations of cholesterol and palmitate with P2, the crystal structure of human P2, after the removal of the fatty acid ligand and water molecules, was used. The cholesterol molecule was constructed using the PRODRG server [39], and the partial charges generated by PRODRG were used after checking. The partial charge of palmitate was added with the VegaZZ server (http://nova.colombo58.unimi.it/vegawe.htm) [40]. The docking simulations were performed both with the AutoDock4 [41] and AutoDock Vina [42] packages, with the aid of the AutoDockTools interface. All the active torsions of the ligands were allowed to rotate freely during conformational sampling. The grid maps were centred on the binding pocket of the protein, with a box size of 29.25×16.5×21 Å. For the AutoDock4 calculations, the Lamarckian genetic algorithm [43] was used, performing 100 runs with 150 individuals in the population, 27000 generations and 5·10^6^ energy evaluations. A cluster analysis was performed and the ligand conformation with the lowest binding energy lay also in the more populated cluster. This conformation corresponded to one of those found by AutoDock Vina and was chosen for further analysis. Structural analysis of the binding modes was made using PyMol, Swiss-PdbViewer [44] and VMD [45]. Swiss-PdbViewer was also used to calculate electrostatic potentials (EP) with the Poisson-Boltzmann method.

### Tryptophan fluorescence analysis of cholesterol binding to P2

Absorbance and fluorescence measurements were done with a Jasco UV-VIS 7850 spectrophotometer and a LS-50 Perkin Elmer spectrofluorimeter, respectively.

The fluorescence spectra of the His-tagged P2 protein, 9.4 µM, in HEPES buffer plus 150 mM NaCl pH 7.5, excited at 280 nm, were recorded as a function of time, in the presence of 10 µM cholesterol. Cholesterol was dissolved in ethanol, and the final concentration of ethanol in the solution was 1% (v/v).

## Results and Discussion

P2 is a peripheral membrane protein of the myelin sheath, and our aim is to combine structural and functional information obtained using the human recombinant P2 protein to elucidate the structure-function relationships in this abundant myelin component. This is an important step towards a more complete understanding of the roles myelin proteins [46] play in the formation and integrity of the myelin sheath, a compact membrane structure crucial to the correct functioning of the vertebrate nervous system.

### Purification of recombinant human P2

From 1 l of autoinduced culture of Rosetta(DE3) cells, 100 mg of pure human P2 were routinely obtained (Figure S1). The importance of cell line selection was highlighted by the fact that using the BL21(DE3) strain, practically no protein could be obtained (data not shown). The sequence of human P2 contains a number of rare codons for *E. coli*, including a total of 7 rare codons for Arg, explaining the crucial need for a modified expression strain. The protein had an apparent molecular weight of 15 kDa, indicating monomeric structure, and it was highly soluble in aqueous buffers. Tryptic peptide mapping by mass spectrometry of a gel band showed that the purified fractions indeed contained human myelin P2 protein (data not shown). Thus, a working large-scale expression and purification system for recombinant human PNS P2 myelin protein was accomplished. This system can be used also in the future for further in-depth studies on P2 structure and function, *e.g.* by site-directed mutagenesis coupled to structure-function assays.

### Conformational analysis by SRCD

**Figure 1:**
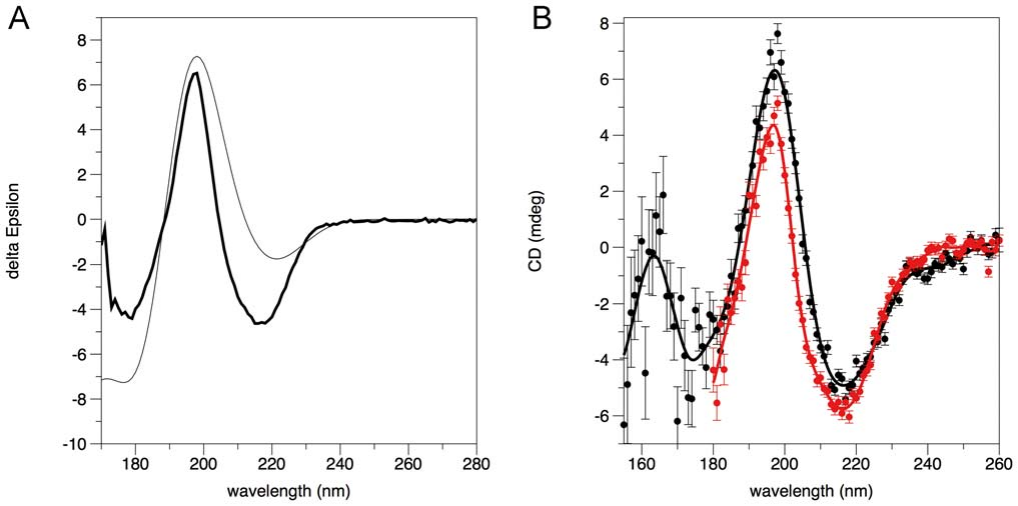
SRCD analysis of human P2 conformation. A. SRCD data for human P2. The SRCD spectrum (thick line) was measured from P2 at ASTRID, Århus. The thin line corresponds to the calculated spectrum based on the human P2 coordinates and the Dichrocalc algorithm. B. Comparison of the SRCD spectra of P2 in solution (red) and as a dried film (black). The amount of protein, as evidenced by the measured absorbance at 190 nm, between the two samples differs by less than 1%. The spectra were collected at the SRCD beamline at BESSY, which has a much lower flux than the CD1 beamline of ASTRID.

The melting point of recombinant human P2 was determined to be +62°C (data not shown), indicating a stable folding of the recombinant protein. SRCD experiments were performed to analyze the folding of human recombinant P2 in solution. The SRCD spectrum of human recombinant P2 (Figure 1A) is consistent with a protein that has a high β-sheet structure content, having negative minima at 216 and 179 nm and a large positive maximum at 198 nm.

The secondary structure content of P2 was analyzed at the DichroWeb server [28]. As expected, the deconvolution indicated a large fraction of beta structure, but different algorithms gave significantly differing solutions. A typical solution with a good fit indicated 17% α-helix, 48% β-sheet, and 35% other structures. The accurate analysis of high-beta folds is a commonly known caveat of CD spectrum deconvolution algorithms. The results do show that recombinant human P2 is folded, as expected, into a mainly β-sheet structure.

Dichrocalc [47] was used to calculate theoretical CD spectra from the experimental atomic coordinates. The SRCD spectrum of human recombinant P2 was then compared with the back-calculated spectrum (Figure 1A). While the overall peak positions agree quite well, the shape of the spectrum is rather different, possibly pointing towards a specific signature of the beta barrel fold of P2, or indicating flaws in the calculation algorithm.

The His-tagged and cleaved P2 were also compared using SRCD. The results show that the His-tagged P2 is folded identically to untagged P2 (data not shown); thus, His-P2 was also used for some of the functional experiments on lipid binding (see below).

SRCD was also used to study P2 in the dry phase, *i.e.* as a film dried onto the surface of the CaF_2_ cuvette. Such measurements on other proteins were recently reported and found to correlate reasonably well with solution data [48]. In the case of P2, the spectra are nearly identical in the region available for solution measurements (Figure 1B), indicating the protein retains its native fold upon the drying procedure. This result further suggests the method could have wider applicability for studying protein conformation at very low vacuum UV (VUV) wavelengths, down to 130 nm. The VUV region of the dried P2 protein shows a strong maximum at 165 nm and minima at 150 and 170 nm.

### Crystal structure

**Figure 2:**
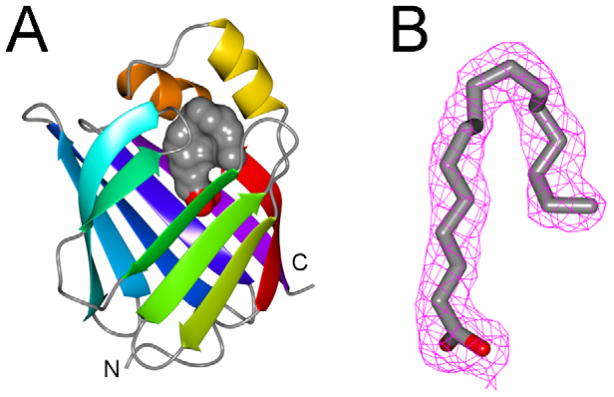
Crystal structure of human P2. A. Overall structure of human myelin protein P2. The bound fatty acid is shown as a solvent-accessible surface. The N and C termini point downwards, and the helical domain is at the top. B. Electron density of the bound fatty acid molecule, which was modeled as a palmitic acid. The map is the final 2F_o_-F_c_ map, contoured at 1 sigma.

**Figure 3:**
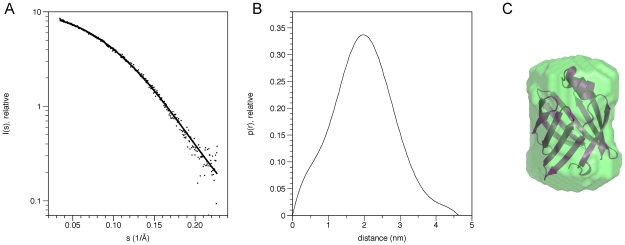
The solution structure of human P2 as analyzed by SAXS. A. The small-angle X-ray scattering curve of human P2, at 4.4 mg/ml. The dots represent the raw data and the curve the fit of the *ab initio* model to the raw data. B. Distance distribution for P2, as calculated by GNOM [65]. C. Superposition of the *ab initio* SAXS solution structure model (green) and high-resolution crystal structure (magenta) of human P2. The solution structure model was built by DAMMIN [66] and DAMAVER [67].

The crystal structures of myelin P2 protein from *Bos taurus* (1PMP, 2.7 Å) and *Equus caballus* (1YIV, 2.1 Å) were previously known [11], [12]. Here, the structure of human recombinant P2 was solved using molecular replacement at 1.85-Å resolution (Table 1, Figure 2A). The similarity to the bovine and equine P2 structures is shown by the rms deviations of Cα positions of 0.60 and 0.68 Å, respectively. The SAXS data further confirm that P2 is monomeric in solution, and its shape in solution is highly similar to that seen in the crystal structure (Figure 3, Table 2).

**Table 2:** Results from SAXS.

	R_g_ (nm)	D_max_ (nm)	Volume (nm^3^)	MW (kDa)*
P2 (SAXS)	1.54	4.5	22.9	11.3
P2 (crystal)**	1.53	4.7	21.1	16.1

*calculated based on a comparison with a calmodulin (17 kDa) model, which has a volume of 34.3 nm^3^.

**calculated from the atomic coordinates of the crystal structure using CRYSOL [65].

The mammalian FABP family includes nine tissue-specific homologues sharing 20–70% amino acid sequence identity [13]. Based on their amino acid sequences, FABPs can be grouped into three groups, which differ in their lipid binding characteristics. The human myelin P2 protein shares the highest amino acid sequence similarities with adipocyte, heart, brain, epidermal, and testis FABP. This group of FABPs can bind fatty acids, retinoids and eicosanoids [13], [49].

**Figure 4:**
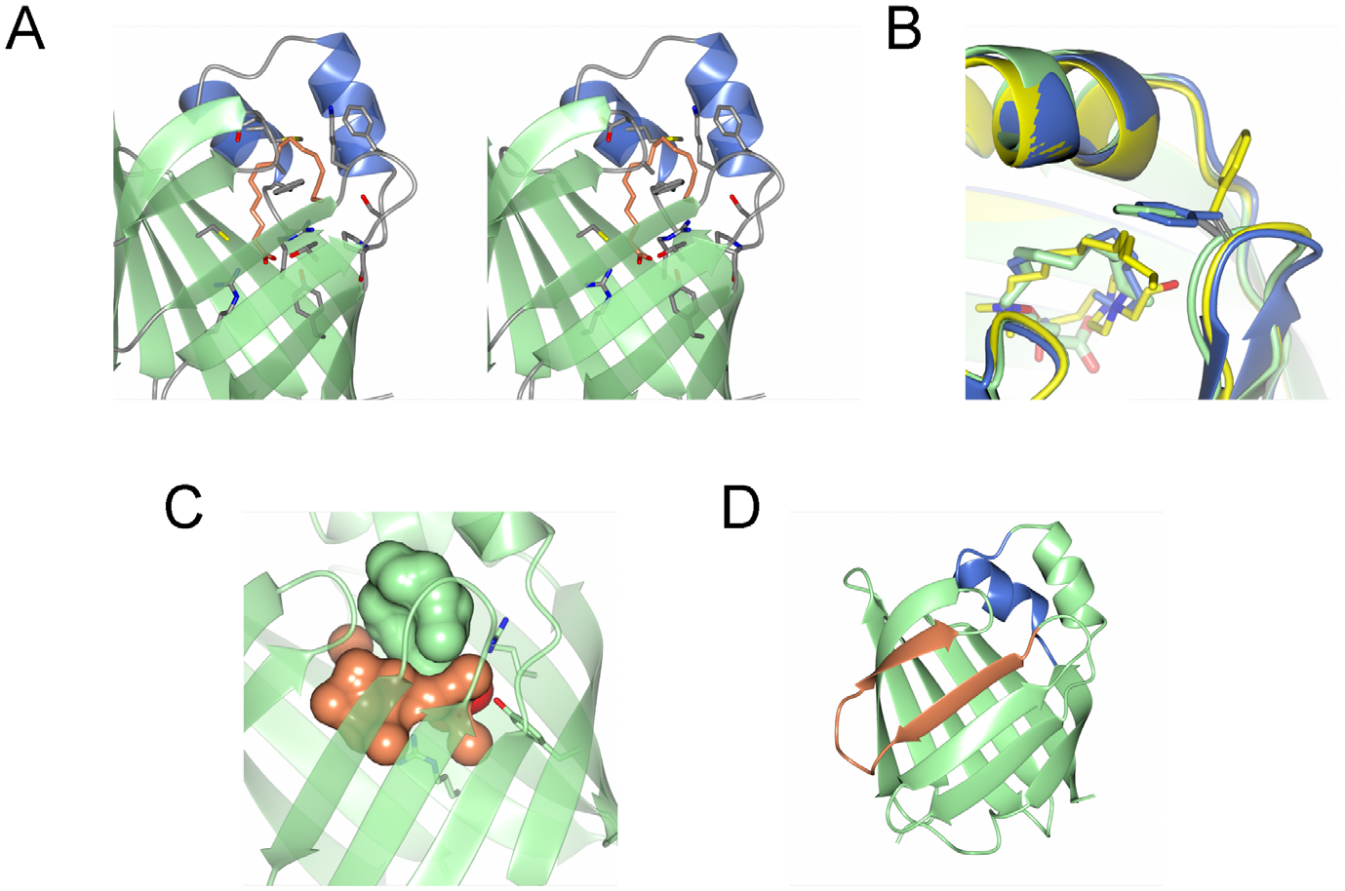
Details from the P2 crystal structure. A. The contacts of the fatty acid molecule and P2, as a stereo view. Note that all hydrophilic contacts are deep in the binding pocket, while the hydrophobic contacts are around the cavity entrance. The helical domain is highlighted in blue. B. Comparison of Phe57 in the available P2 protein structures (human, green; bovine chain B, blue; equine, yellow). *Via* a rotation of the side chain, more space can be made for larger ligands. C. In addition to the fatty acid (green/red), the P2 cavity is also filled by water (orange), indicating the possibility of fitting larger and chemically variant ligands. Most of these water molecules are hydrogen bonded to the inner walls of the cavity. D. Mapping of potential disease-linked epitopes onto human P2. The EAN epitope (residues 58–73) is highlighted in orange, and the epitope recognized by GBS patients (residues 14–25) in blue.

The human P2 crystal structure is highly similar to that seen in other FABP members: 10 anti-parallel β-strands (referred to as strands A–J in the discussion below), forming two nearly orthogonal β-sheets that form an elliptical twisted β-barrel [50], [51]. The first two strands are linked together by two α-helices, which close the barrel from one end. In FABPs, the fatty acid is bound inside the barrel carboxyl head first [52]; while the hydrophobic contacts are around the cavity entrance, hydrophilic contacts are deep in the binding pocket (Figure 4A). The ligand binding cavity extends from the helix-turn-helix motif, which functions as a portal region for fatty acids. It is assumed that a conformational change of the portal region occurs during fatty acid binding or release [53]. The portal region may bind to membranes, and this binding could catalyze a conformational change. Some FABPs are cytosolic, only transferring fatty acids in the cytosol, but P2 is an extrinsic membrane protein between myelin membranes and possibly able to transfer fatty acids *via* a direct interaction with membranes.

In the human P2 crystal structure, a palmitate molecule, well-defined in the electron density (Figure 2B,4A), was modeled into the lipid-binding cavity in a bent conformation, superimposing the position and orientation of oleate in the bovine P2 structure [11]. The electron density of the bound ligand clearly indicated the presence of a carboxyl group and a long aliphatic tail, very well fitting a 16-carbon chain. Since no lipid ligands were added to the sample at any stage of purification, the ligand must come from the *E. coli* cells used for protein expression; palmitate being the most abundant fatty acid in *E. coli* [54] also makes it a likely ligand for recombinant P2. The carboxyl group of the ligand points inside the barrel, closely interacting by means of H-bonds and salt bridges with the side chains of Arg126, Arg106, and Tyr128, the three residues totally buried into the inner cavity of the barrel that are important for the binding of fatty acids and retinoids in the FABP family [51], [55]; these residues provide an overall basic feature to the binding site surface.

A comparison between the three currently available crystal structures for P2 indicates a conformational change at the mouth of the lipid-binding cavity, involving Phe57, which lies in a loop between β-strands C and D (Figure 4B). In human P2 and one chain of the bovine P2 structure, it is closed on top of the cavity, while in equine P2 and the remaining chains in the bovine P2 crystal, it is in an open conformation. A role for this residue in the opening of the cavity and lipid binding has also been suggested before [10], [56]; it seems to be important for the formation of a fatty-acid – FABP complex, and its mutation results in a loss of fatty acid binding. The closed position of Phe57 in human P2 allows it to become in contact with the ligand present in the binding pocket.

The presence of several well-ordered water molecules (Figure 4C) inside the P2 cavity indicates that also larger, at least partly more hydrophilic, ligands could be accommodated in this space. A conserved structural water molecule in the FABP family has also been reported; P2 was not analyzed in that study [57]. This water is located in a pocket close to the protein external surface, in a wedge formed by the loop between β-strands D and E. From the human P2 structure, it is evident that this water molecule is also present, being H-bonded to the backbone NH group of Val84 and the carbonyl oxygens of Lys65 and Gln68 (data not shown). It was suggested that such a tightly bound water molecule could stabilize the βD-βE loop [57]. Interestingly, main-chain hydrogen bonds are lacking between βD and βE; therefore, a more important structural role could be hypothesized for this water molecule, which could help to keep together the walls of P2 across this structural gap.

The main autoimmune epitope of P2 locates at residues 58–73 [58], and a peptide corresponding to this region has been been used extensively to induce and study the animal model of GBS, EAN. The minimal T cell epitope of P2 includes a subset of this region, residues 61–70 [59]. In EAN, a 16-mer of the P2(58–73) peptide has also been used for treatment and vaccination [60]. At the structural level (Figure 4D), the epitope localizes to a β-strand-turn-β-strand unit, on the side opposite to the helical domain. In fact, these are the strands βD and βE, between which no main-chain hydrogen bonds exist. In addition, the epitope recognized by GBS patients in a recent study [20] comprises residues 14–25, which locates at the helical subdomain.

### Potential membrane binding sites

**Figure 5:**
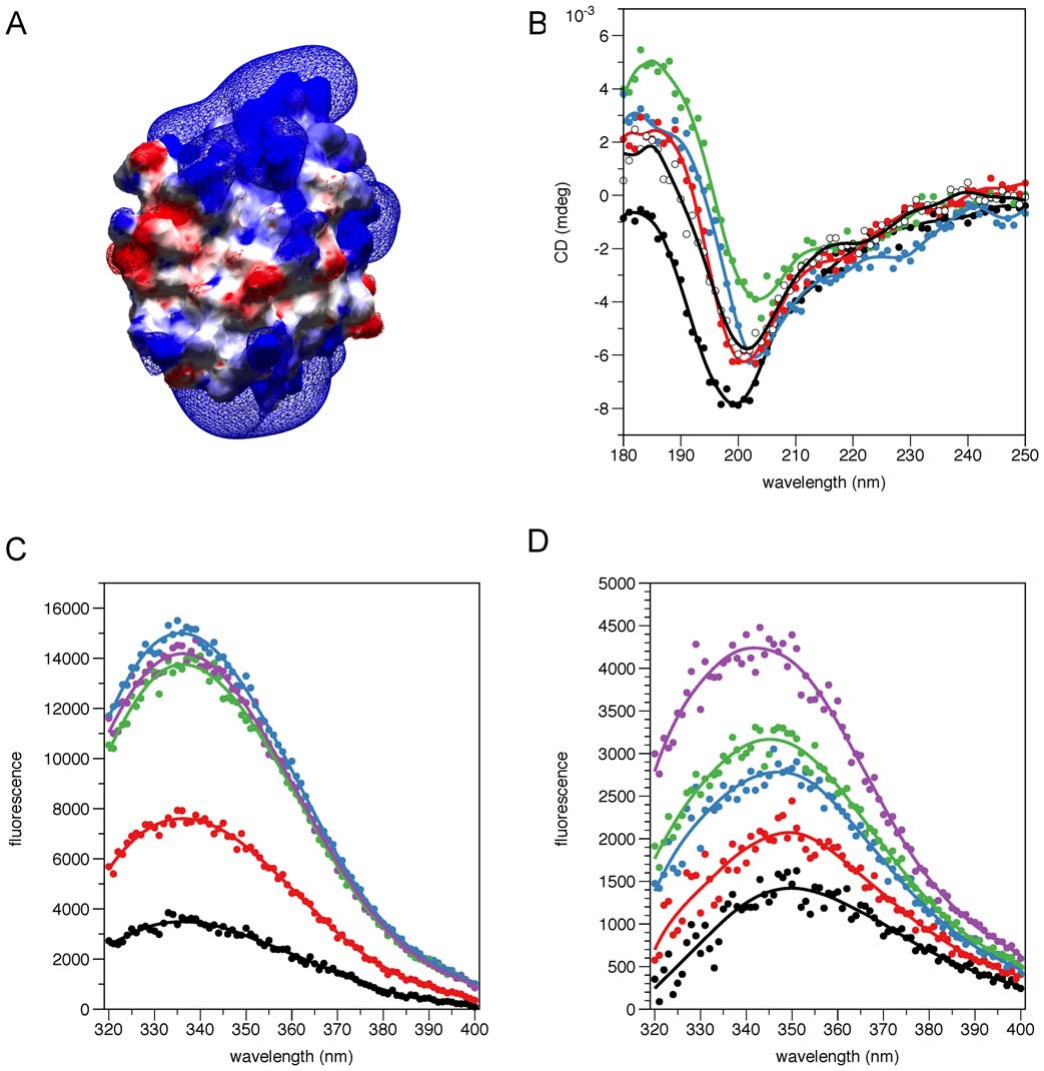
Possible membrane binding regions. A. Electrostatic potential of human P2; the view is the same as in Figure 2A. Note the positively charged faces at the top and bottom. For the electrostatic potential map, shown as a mesh, cutoffs of −2.3 *k*b*T*/e (red) and +2.3 *k*b*T*/e (blue) were used. The protein is visualized with its solvent-accessible surface, onto which the EP is mapped. B. SRCD analysis of the conformation of the N-terminal peptide from P2 in the presence of different membrane-mimicking compounds. Black, buffer; green, 0.5% DPC; blue, 0.5% SDS; red, 30% TFE; white, 0.5% DMAO. C. Titration of the P2 N-terminal peptide with DPC, followed by intrinsic Trp fluorescence. The DPC concentrations were 0.1 (black), 0.2 (red), 0.5 (blue), 1 (green), and 2 (magenta) %. Difference spectra are shown, *i.e.* the emission spectrum in the absence of DPC has been subtracted. D. Titration of P2 with DPC, followed by intrinsic Trp fluorescence. Colouring of the difference spectra as in C. The titration of His-P2 is shown in Figure S3.

The EP surface of the highly basic human P2 shows two large opposite highly positive regions separated by an almost neutral rim (Figure 5A). This charge distribution is shared by all the known P2 protein structures, and therefore, could be important for the interaction of P2 with two apposing membrane leaflets of myelin. This suggests a structural “glue” role for P2, analogous to that of MBP in the CNS. Supporting this hypothesis, all the cytosolic FABPs show a relatively featureless neutral, or slightly negative, EP surface (data not shown), in spite of the overall structural features they have in common with P2 proteins.

As another means of identifying membrane-binding segments, we used sequence analysis in Amphipaseek [31]. The region highlighted by the algorithm contains the N-terminus of P2, residues 1 to 10 (SNKFLGTWKL), lying in one of the positively charged surface areas discussed above. In the crystal structure (Figure S2), this segment is bound to the protein surface, forming a short 1-turn 3_10_-helix, a structural feature strongly conserved in the group of FABSs highly similar to P2, and an extended fragment. The Trp residue is actually anchored under the C-terminus of the protein. A part of the interaction is also formed by the Met encoded by the start codon, which should be absent in the native protein. Thus, the conformation of the very N terminus is slightly different between the native bovine/equine and recombinant human proteins. We synthesized a peptide corresponding to this putative membrane binding region and studied its conformation upon binding to membrane mimics, such as DPC micelles, using SRCD (Figure 5B). While the peptide had no significant secondary structure in phosphate buffer, it showed strong helical character in all the tested membrane-mimicking conditions, including 0.5% DPC, 0.5% SDS, 0.5% DMAO, and 30% TFE. Thus, at least outside the context of the full-length P2 protein, this N-terminal sequence has a tendency to associate with membrane mimics.

The interaction with DPC was also probed by fluorescence spectroscopy, using the intrinsic signal from the Trp residue within the peptide (Figure 5C). The results indicate that the environment of the Trp residue in the peptide changes upon increasing concentrations of DPC micelles. The difference peak is centered at 337 nm, and reaches a plateau at 0.5% DPC. These results indicate direct binding of the peptide to DPC micelles. Using full-length P2, both His-tagged and untagged, a fluorescence signal was also induced by DPC in a concentration-dependent manner (Figure 5D,S3). However, whether this is a result of the N-terminal segment binding to DPC micelles, of the binding of monomeric DPC inside P2, or both, remains a subject for future studies.

### Binding of lipids to human P2: molecular docking simulations and fluorescence spectroscopy measurements

The crystal structure of human P2 was used as a starting point for lipid docking simulations, in order to investigate if the lipid constituents of myelin could be bound by P2. While it has been postulated that most of the major lipids found in myelin are much larger than the binding pocket in P2 [12], this is not true for cholesterol, which takes up a volume of 376 Å^3^ in its fully extended conformation with all hydrogen atoms added. The volume of the P2 binding pocket is 976.4 Å^3^ (as calculated by CASTp [61]). In addition, also some other phospholipids, such as dimyristoyl phosphatidyl ethanolamine (DMPE) could fit into the P2 binding pocket (the volume of a bent conformation, as obtained by preliminary docking measurements, has a volume of 649 Å^3^). However, the best candidate was cholesterol, one of the most abundant lipids in myelin, which has a polar head that fits very well to the electrostatic characteristics of the binding site.

**Figure 6:**
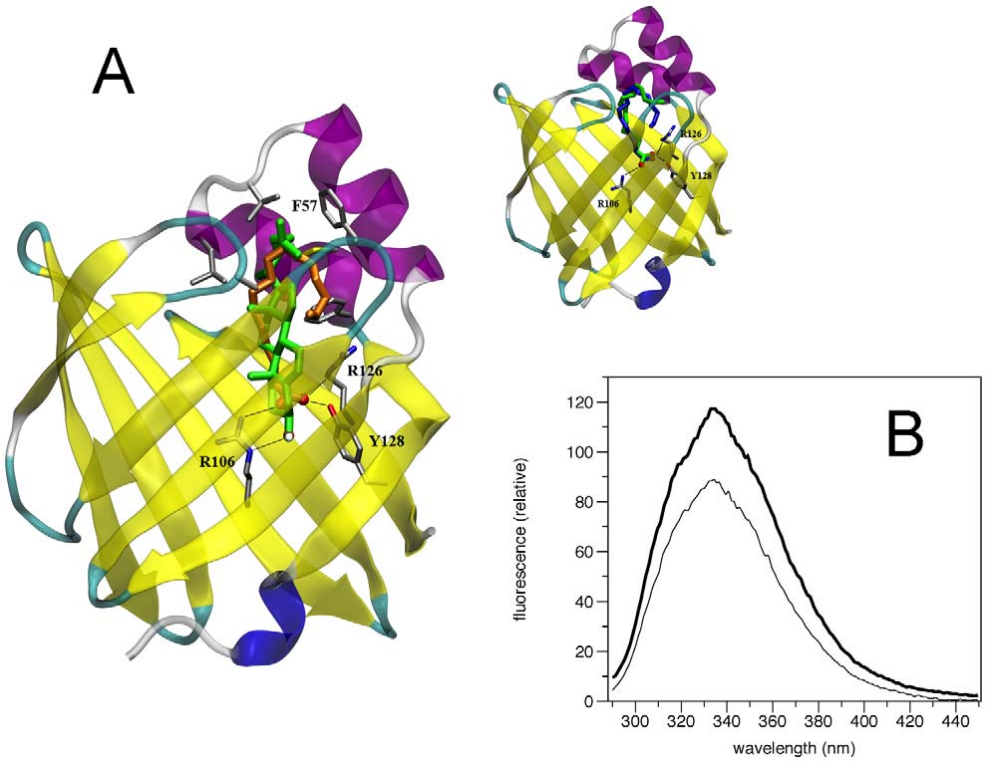
Identification of cholesterol as a potential ligand for P2. A. Structure of the more favourable complex of P2 protein with cholesterol (green), obtained by the docking simulations. The position of the palmitate (orange) found in the crystal is shown for comparison. The polar heads of the two ligands are coloured by element. The favourable interactions formed at the binding site with Arg106, Arg126, and Tyr128 residues (in sticks) are shown as black lines. The main hydrophobic contact residues are also shown. Inset: Structure of the more favourable complex of P2 with palmitate (green), obtained by the docking simulations. The position of the palmitate (blue) as found in the crystal is shown also. Arg106, Arg126, and Tyr128 and their favourable interactions with the two ligands are shown. B. Emission fluorescence spectra of a) human P2 9.4 µM and b) human P2 9.4 µM plus cholesterol 10 µM after 120 min of incubation at room temperature.

The docking simulations, both with AutoDock4 and AutoDock Vina, converged to a structure that shows the ligand inside the binding cavity in the same position as the crystallographically detected ligand palmitate. The cholesterol molecule has its polar region oriented towards the conserved Arg106-Arg126-Tyr128 (RRY) binding site (Figure 6A), forming a H-bond with Arg106. For comparison and validation of the docking algorithms, docking simulations were performed also with palmitate. The results show that the ligand position and conformation are the same as in the crystal structure, with exactly the same kind of interaction with the RRY residues (Figure 6A, inset). The docking experiments indicated cholesterol as a candidate for binding, with an affinity computed of the same order of magnitude as palmitate, as suggested by the respective estimated values for the free energy of binding (data not shown).

Recently, it has been observed that the beta-barrel Niemann-Pick Type C2 protein (NPC2), which plays a role in late endosomal/lysosomal transport of cholesterol, shows a time dependent decrease of its fluorescence when interacting with cholesterol molecules [62]. Thus, we further probed the interaction between human P2 and cholesterol using intrinsic Trp fluorescence. The binding of cholesterol to P2 was monitored by observing the protein fluorescence quenching induced by stoichiometric amounts of ligand. P2, at 9.4 µM, showed a slow decrease of fluorescence intensity as a function of time, in the presence of 10 µM cholesterol, that reached a stable value after 120 min at room temperature. The presence of cholesterol, therefore, involves a decrease of about 25% of fluorescence intensity of the protein (Figure 6B), which is not detectable when ethanol alone was added. Since an analogous decrease of human P2 protein intrinsic fluorescence was observed as was seen for NPC2 [62], [63], we suggest that the binding of cholesterol causes the quenching of fluorescence from the P2 protein. This interpretation is well supported by the docking results, showing a favourable binding of cholesterol into the barrel pocket, with average distances between Trp aromatic rings and cholesterol ranging from 7 to 9 Å.

While our experiments indicate cholesterol binding by P2, at the moment, it is unclear whether the bound cholesterol would originate from the plasma membrane or from free cholesterol. In this respect, it is interesting to note that when P2 is purified from tissue in a lipid-bound form, the major lipid present in the protein preparation is cholesterol [64]. Since P2 also is localized to compact myelin, it is likely to bind to the membrane surface at cholesterol-rich microdomains. In future studies, we aim to investigate whether P2 is able to bind cholesterol from vesicles, in experiments analogous to those reported for NPC2 [62], [63].

### Concluding remarks

We used a recombinant form of human myelin P2 protein to derive high-resolution structural data on this highly abundant PNS peripheral membrane protein. The crystal structure allows a better understanding of potential disease-related epitopes, and provides clues to the functional mechanisms of P2 in lipid transport and myelin membrane stabilization. P2 may bind myelin lipids, such as cholesterol, with high affinity, and it could interact with two apposing cytoplasmic leaflets of the multilayered myelin membrane simultaneously. P2 may, thus, partially share functions with MBP, another molecule suggested to act as ‘molecular glue’ in myelin.

## Supporting Information

Figure S1 Purification of recombinant human P2. Top, gel filtration of purified P2 results in a single peak. Bottom, SDS-PAGE analysis of the fractions from the gel filtration peak indicates the expected molecular weight.
(0.35 MB DOC)

Figure S2 The N-terminal region of P2. The N terminus is shown in blue and the C terminus in red. Side chains are shown for residues 1–10, as well as the N-terminal Met residue. This region corresponds to the putative membrane-binding peptide used in the assays.
(0.43 MB DOC)

Figure S3 Titration of His-P2 with DPC, followed by intrinsic Trp fluorescence. The colouring and other details as in Figure 5C, 5D.
(0.15 MB DOC)
