# Supplementary material for: Structural and Functional Characterization of Human Peripheral Nervous System Myelin Protein P2
Source: PLoS One. 2010 Apr 22;5(4):e10300. doi: 10.1371/journal.pone.0010300 (PMC2858655; doi:10.1371/journal.pone.0010300)
Supplement: Figure S1 — Purification of recombinant human P2. Top, gel filtration of purified P2 results in a single peak. Bottom, SDS-PAGE analysis of the fractions from the gel filtration peak indicates the expected molecular weight. (0.35 MB DOC) [file pone.0010300.s001.doc]

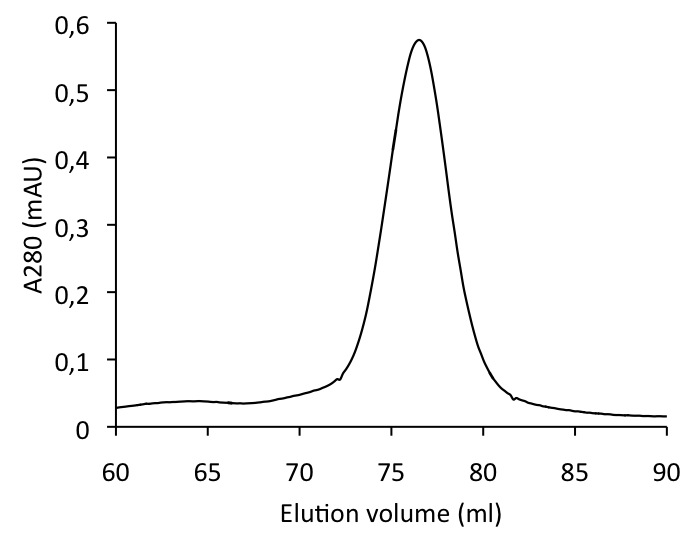


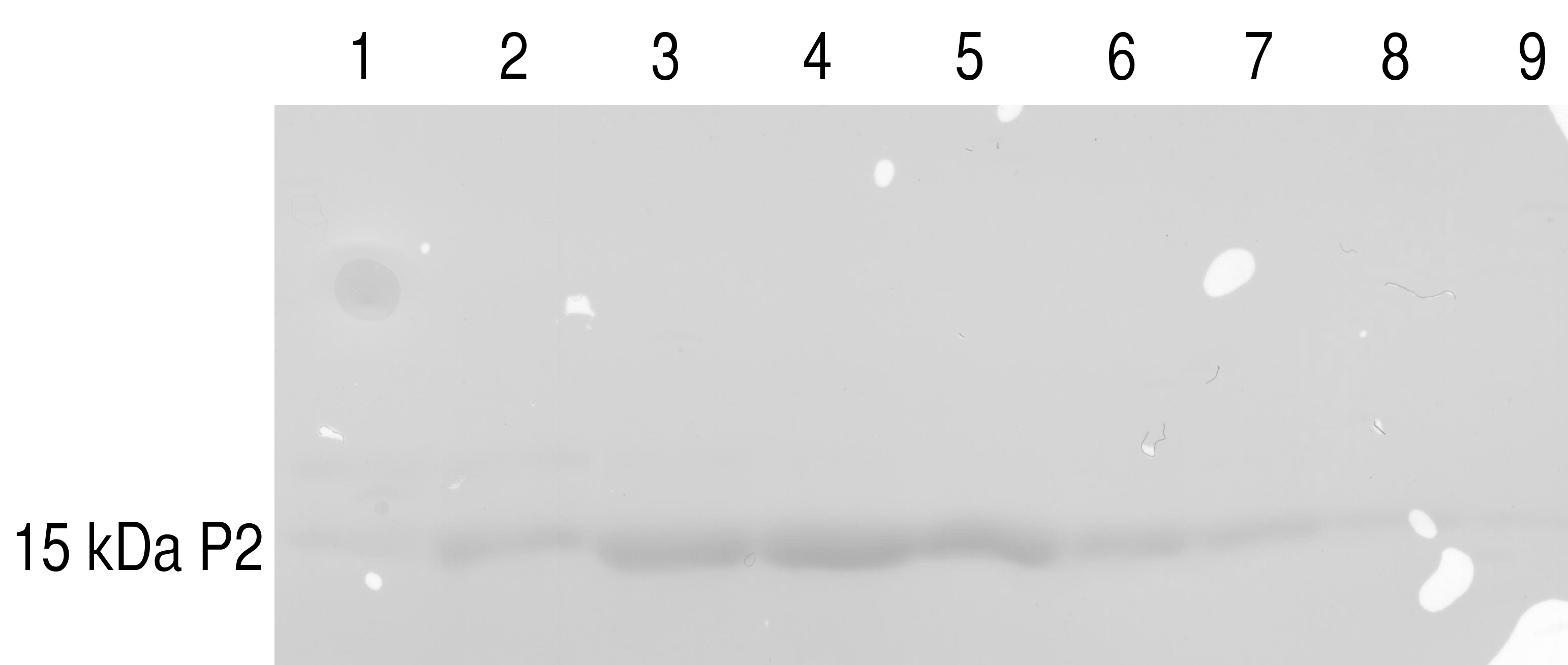


*Figure S1. Purification of recombinant human P2.*

Top, gel filtration of purified P2 results in a single peak. Bottom, SDS-PAGE analysis of the fractions from the gel filtration peak indicates the expected molecular weight.
