# Supplementary material for: Structural and Functional Characterization of Human Peripheral Nervous System Myelin Protein P2
Source: PLoS One. 2010 Apr 22;5(4):e10300. doi: 10.1371/journal.pone.0010300 (PMC2858655; doi:10.1371/journal.pone.0010300)
Supplement: Figure S2 — The N-terminal region of P2. The N terminus is shown in blue and the C terminus in red. Side chains are shown for residues 1–10, as well as the N-terminal Met residue. This region corresponds to the putative membrane-binding peptide used in the assays. (0.43 MB DOC) [file pone.0010300.s002.doc]

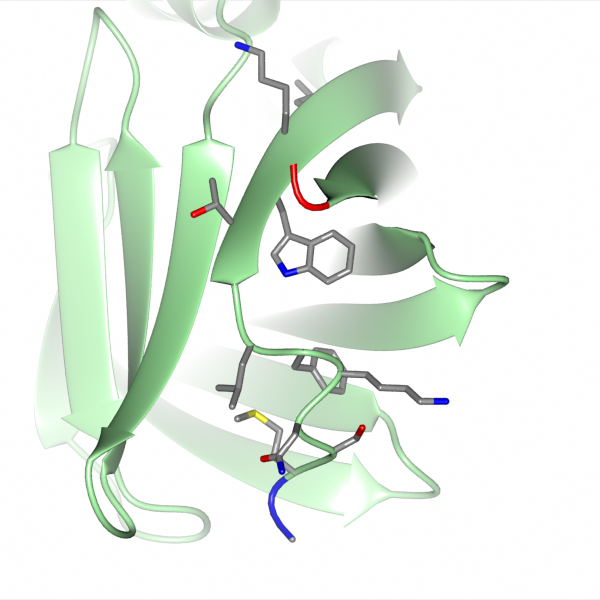


*Figure S2. The N-terminal region of P2.*

The N terminus is shown in blue and the C terminus in red. Side chains are shown for residues 1-10, as well as the N-terminal Met residue. This region corresponds to the putative membrane-binding peptide used in the assays.
