# Supplementary material for: Structural and Functional Characterization of Human Peripheral Nervous System Myelin Protein P2
Source: PLoS One. 2010 Apr 22;5(4):e10300. doi: 10.1371/journal.pone.0010300 (PMC2858655; doi:10.1371/journal.pone.0010300)
Supplement: Figure S3 — Titration of His-P2 with DPC, followed by intrinsic Trp fluorescence. The colouring and other details as in Figure 5C, 5D. (0.15 MB DOC) [file pone.0010300.s003.doc]

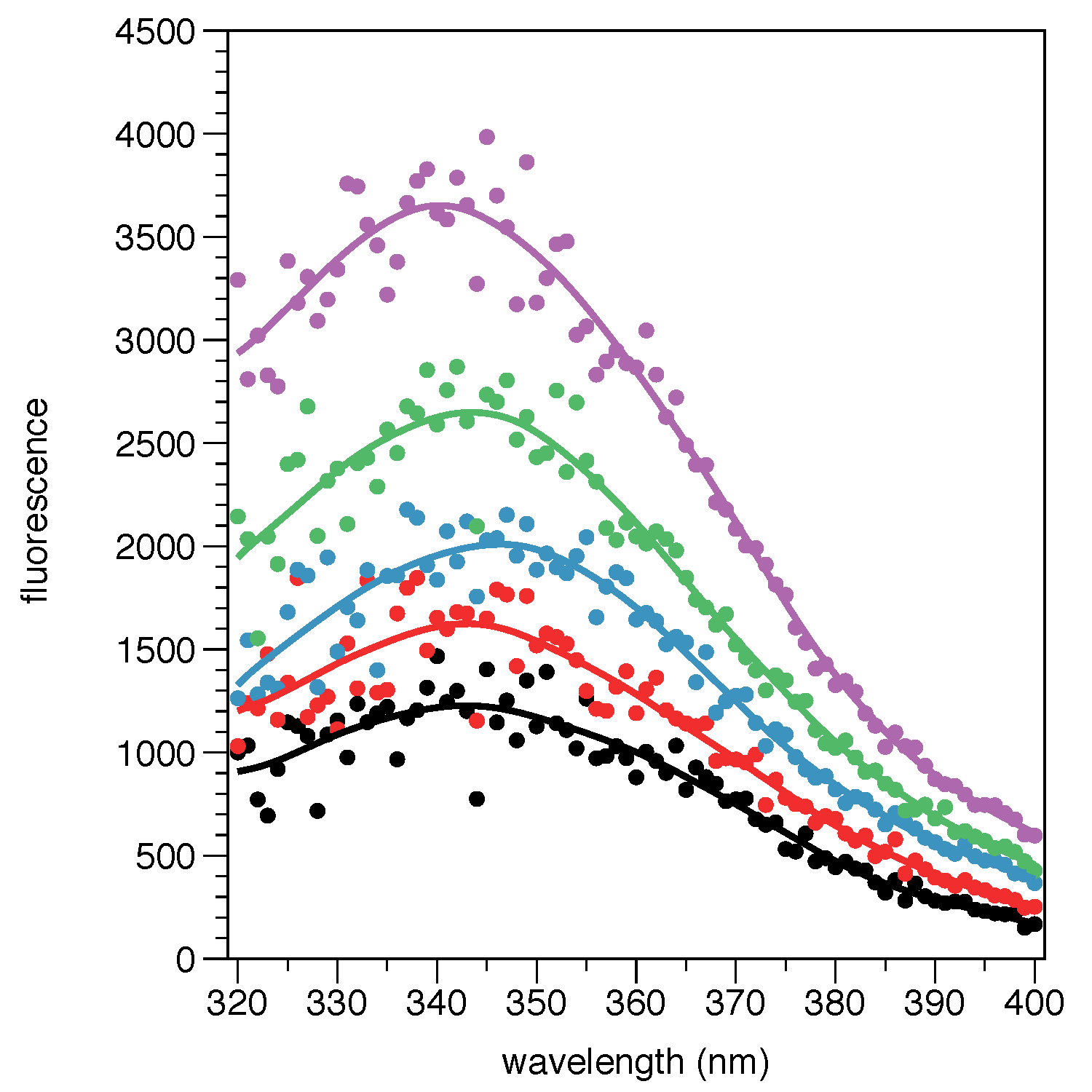


*Figure S3. Titration of His-P2 with DPC, followed by intrinsic Trp fluorescence.*

The colouring and other details as in Figure 5C,5D.
